# An autopsy case of idiopathic fulminant eosinophilic myocarditis: a case report

**DOI:** 10.1093/ehjcr/ytaf262

**Published:** 2025-05-26

**Authors:** Eiji Koyama, Masashi Yamaguchi, Hiroshi Koyama, Shinichi Teshima, Shigeru Saito

**Affiliations:** Department of Cardiology, Shonan Kamakura General Hospital, 1370-1 Okamoto, Kamakura, Kanagawa 247-8533, Japan; Department of Cardiology, Shonan Kamakura General Hospital, 1370-1 Okamoto, Kamakura, Kanagawa 247-8533, Japan; Department of Critical Care Medicine, Shonan Kamakura General Hospital, 1370-1 Okamoto, Kamakura 247-8533, Japan; Department of Pathology and Diagnostics, Shonan Kamakura General Hospital, 1370-1 Okamoto, Kamakura 247-8533, Japan; Department of Cardiology, Shonan Kamakura General Hospital, 1370-1 Okamoto, Kamakura, Kanagawa 247-8533, Japan

**Keywords:** Eosinophilic myocarditis, Cardiogenic shock, Mechanical circulatory support, Right ventricular dysfunction, Autopsy, Case report

## Abstract

**Background:**

Eosinophilic myocarditis is a rare and fatal form of inflammatory myocardial disease. It is frequently caused by a systemic disorder, which can remain undetected in up to one-third of patients. Eosinophilic myocarditis can have mild to fulminant clinical presentation.

**Case summary:**

We present a case of fulminant eosinophilic myocarditis in a 68-year-old woman. The patient was admitted for cardiogenic shock with electrocardiographic abnormalities and elevated troponin I levels. After an unremarkable coronary angiography, a myocardial biopsy was performed. Right-heart catheterization revealed low cardiac output and elevated mean pulmonary arterial wedge pressure. A lower pulmonary artery pulsatility index (0.63) indicated right ventricular dysfunction. Despite intensive treatment including dobutamine, the patient suffered a cardiac arrest triggered by sustained ventricular tachycardia. The patient was successfully resuscitated using veno-arterial extracorporeal membrane oxygenation and intra-aortic balloon pumping (IABP). IABP was upgraded to Impella CP at our hospital after the patient could not maintain her blood pressure. A biopsy showed eosinophilic myocarditis, despite a stagnant peripheral blood eosinophil count. Initiating methylprednisolone and immunoglobulin therapies improved left ventricular function. However, right ventricular function did not improve. Despite these treatments, the patient died 31 days after admission. Autopsy revealed minimal infiltration of inflammatory cells, suggesting the effectiveness of this medication. However, extensive necrotic changes were observed in the myocardium.

**Discussion:**

This case involved fulminant eosinophilic myocarditis with biventricular dysfunction treated with mechanical circulatory support and steroid therapy. Prompt steroid treatment before confirmation of biopsy results may improve the prognosis.

**Learning points**

- Myocardial biopsy remains important in cases of fulminant myocarditis because systemic clues to the underlying mechanism causing myocardial inflammation may be absent.
- Fulminant myocarditis presenting with right heart failure has a poor prognosis and is difficult to treat.
- If fulminant myocarditis is suspected, treatment with steroid therapy should be initiated early after myocardial biopsy.

## Introduction

Eosinophilic myocarditis (EM) is a rare inflammatory myocardial disease and accounts for approximately 15% of cases of fulminant myocarditis.^1^ EM is associated with eosinophilic clinical conditions, including hypersensitivity reactions, hypereosinophilic syndrome, granulomatosis with polyangiitis, parasitic infections, drug reactions, and malignancies; however, the aetiology is not always apparent. In a report involving 179 patients, 35.7% of those with histologically proven EM lacked a definitive aetiology.^2^

EM may be mildly symptomatic or fulminant. Additionally, the in-hospital prognosis is generally poor, with an in-hospital mortality rate of approximately 22.3%.^2,3,4^

We present an autopsy case of a patient with cardiogenic shock who was treated with veno-arterial extracorporeal membrane oxygenation (VA-ECMO), concomitant Impella support (ECPELLA), and corticosteroids for biopsy-confirmed EM.

## Summary figure

**Figure:**
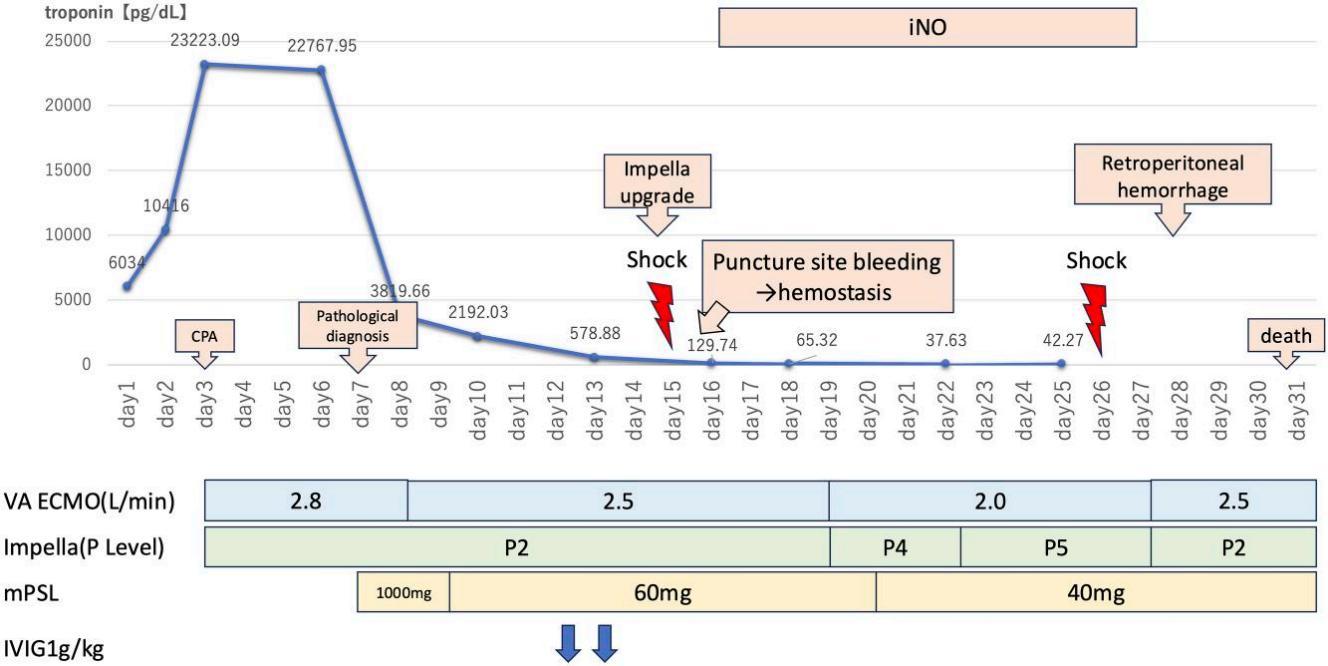


## Case presentation

**Figure 1:**
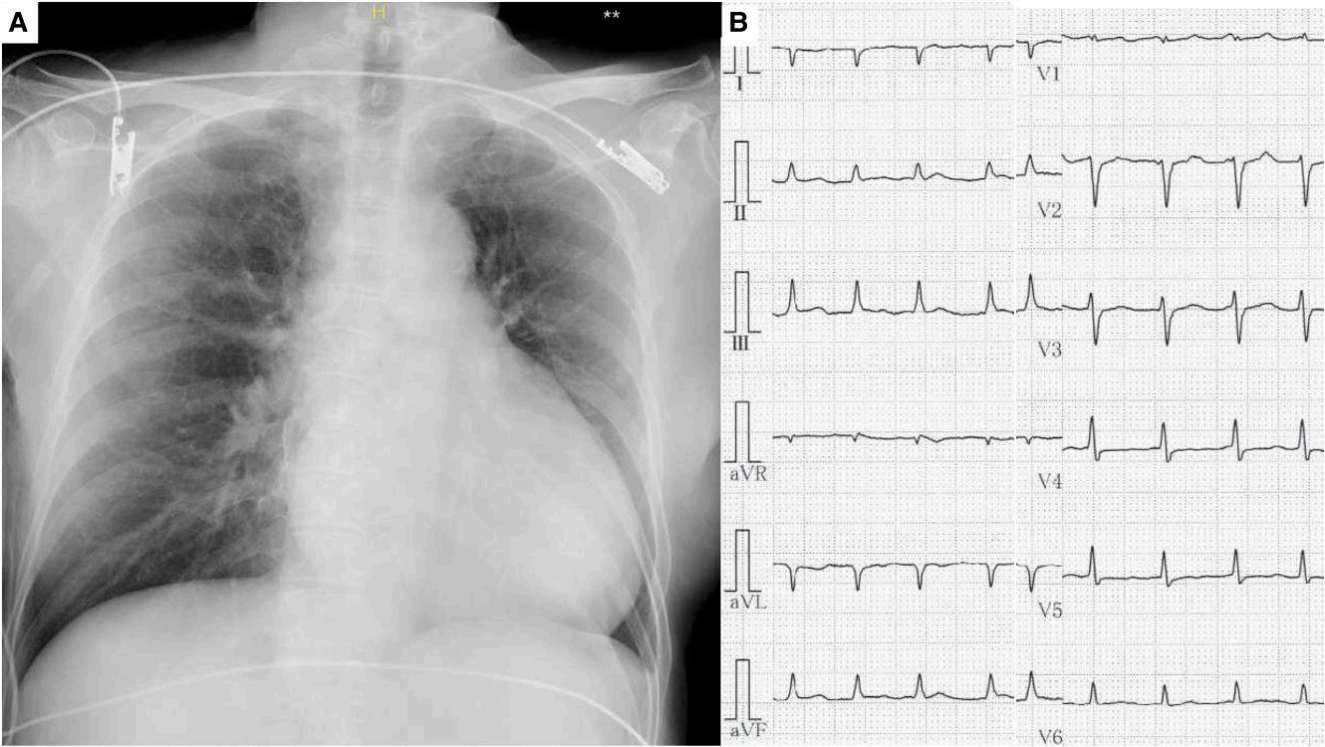
Chest radiography and electrocardiography (ECG). Chest radiography did not reveal pulmonary oedema or pleural effusions. (*A*) ECG revealed atrial fibrillation, low voltage, small QRS voltage, and non-specific repolarization abnormalities (*B*).

A 68-year-old female with hypertension due to primary aldosteronism over the past three years was treated with esaxerenone. Her blood pressure decreased several weeks before admission. She presented with anorexia and nausea, culminating in cardiogenic shock and emergency admission. On admission, her blood pressure was 88/48 mmHg, with an irregular pulse rate of 110/min and signs of peripheral circulatory impairment. The oxygen partial pressure was maintained at 67.5 mmHg with 3 L/min oxygen via a nose cannula. Her serum lactate level was slightly elevated at 2.3 mmol/L. Blood tests revealed elevated troponin I (6034 pg/mL; reference, <45.2 pg/mL) levels and N-terminal prohormone of brain natriuretic peptide (>35 000 pg/mL; reference, <125 pg/mL) levels, while creatinine level (0.99 mg/dL; reference, 0.65–1.07 mg/dL) and eosinophil count (0.3 × 109/L; reference, 0.7 × 109/L) were normal (*Table 1*). Chest radiography revealed no signs of pulmonary oedema or pleural effusion. Electrocardiography revealed atrial fibrillation, low voltage, and non-specific repolarization abnormalities (*Figure 1*). Transthoracic echocardiography showed decreased left ventricular ejection fraction (LVEF) to 40%, oedematous and slightly thickened LV wall (14 mm), and minor pericardial effusion without signs of tamponade (see Supplementary material online, *Video S1*). Right ventricular (RV) function remained stable with no RV load.

**Table 1:** Laboratory test results and reference values at admission

Test	Result	Reference value
Haemoglobin	12.2 g/dL	13.5–16.9 g/dL
White blood cell count	9.4 × 10^9^/L	3.9–10.9 × 10^9^/L
% Neutrophils	87.4%	41–71%
% Eosinophils	3.6%	1–8%
% Lymphocytes	4.8%	19–48%
% Monocytes	4.0%	5–15%
CRP	61.5 mg/L	<5 mg/L
Urea	38.8 mg/dL	<49 mg/dL
Cre	0.99 mg/dL	0.67–1.17 mg/dL
Troponin I	6034pg/mL	＜26.2 pg/mL
NT-pro BNP	35 000 > ng/L	<172 ng/L
Lactate	2.3 mmol/L	0.5–2 mmol/L

CRP, C-reactive protein; Cre, creatinine; NT-pro BNP, N-terminal prohormone of brain natriuretic peptide.

Emergency coronary angiography revealed no significant coronary stenosis or obstructions. Right heart catheterization demonstrated a low cardiac index of 1.54 L/min/m^2^ with no elevated mean pulmonary capillary wedge pressure (14 mm Hg) or mean right atrium pressure (11 mmHg), and no significant pulmonary hypertension (pulmonary artery 23/16, mean 14 mmHg). A lowered pulmonary artery pulsatility index [(systolic pulmonary artery pressure—diastolic pulmonary artery pressure)/right atrial pressure] of 0.63 indicated the coexistence of RV dysfunction. An endomyocardial biopsy of the RV septum was performed. The patient, suspected of myocarditis with persistent cardiogenic shock (Society for Cardiovascular Angiography and Interventions, stage C), was immediately started on amiodarone and dobutamine under close monitoring. On day 3, sustained ventricular tachycardia (VT) caused cardiopulmonary arrest. Despite resuscitation with VA-ECMO and intra-aortic balloon pumping, the circulatory insufficiency persisted, and she was transferred to our hospital for an Impella CP upgrade.

**Figure 2:**
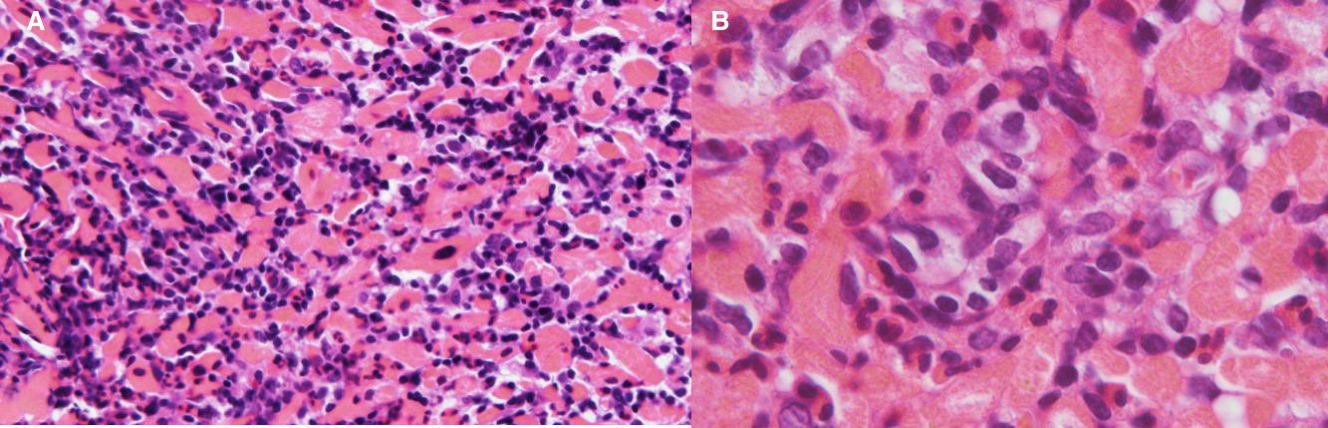
Histopathological analysis of a biopsy specimen of the myocardium showing infiltration with inflammatory cells, mainly eosinophils. (*A)* Haematoxylin and eosin staining, × 20 magnification; (*B)* Haematoxylin and eosin staining, × 100 magnification).

On admission, her blood pressure was 95/62 mmHg, pulse rate was 78/min, and body temperature was 35.8°C with VA-ECMO at 2.8 L/min, Impella CP at 1.2 L/min, noradrenaline at 0.3 μg/kg/min, and dobutamine at 3 μg/kg/min. On day 7, pathological staining of the RV sample revealed significant eosinophilic infiltration, consistent with EM (*Figure 2*). The tests for serum anti-cytoplasmic and antinuclear antibodies and screening for parasites returned negative results. Furthermore, the eosinophil count did not increase during hospitalization. Corticosteroid pulse therapy (methylprednisolone 1000 mg/day for 3 days) and corticosteroid (methylprednisolone 1 mg/kg/day for 2 weeks) were administrated, with intravenous immunoglobulin therapy (1 g/kg for 2 days).

By the 12th day of treatment, LVEF improved from 9.4% to 32.1%, myocardial oedema reduced, and troponin I level reduced from 23 000 pg/mL to 31.69 pg/mL. Despite these improvements, the central venous pressure and mean pulmonary artery pressure remained high (16 and 18 mmHg, respectively), and RV dysfunction was present (see Supplementary material online, *Video S2*). Therefore, inhaled nitric oxide was administered.

On day 15, her condition did not improve, and Impella CP withdrawal was difficult. Therefore, Impella 5.5 was inserted through the right subclavian artery for long-term management in the hope of a bridge to recovering cardiac function. However, haemorrhagic shock occurred on day 26, revealed by contrast-enhanced computed tomography showing active retroperitoneal haemorrhage. The patient died on day 31 despite the best supportive care.

**Figure 3:**
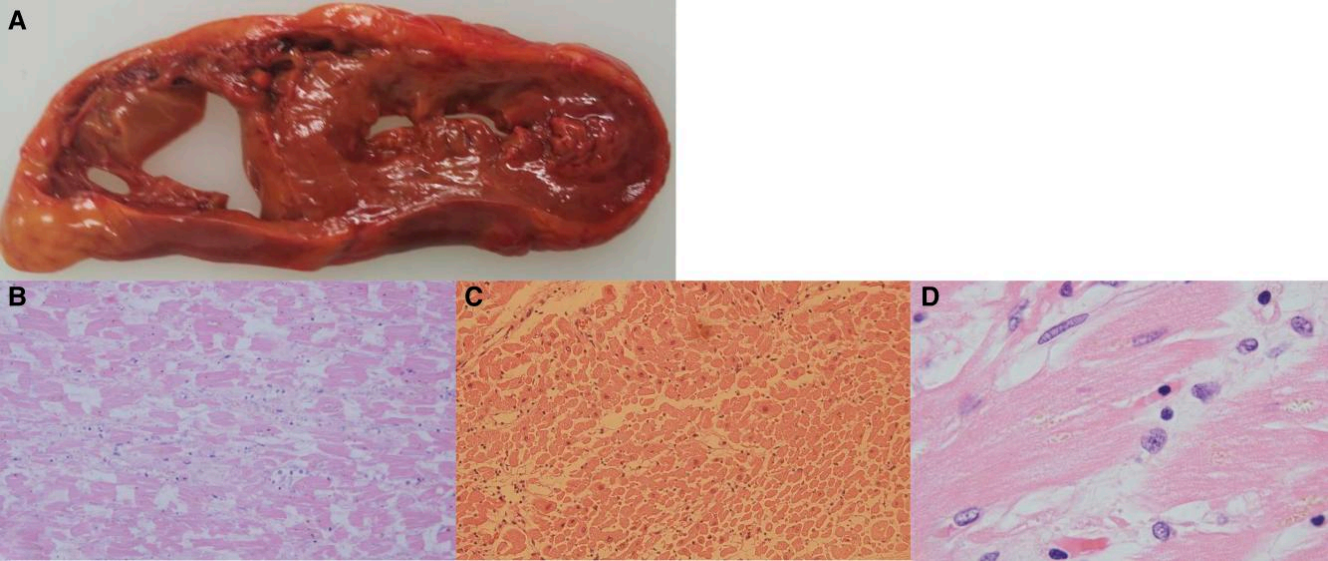
Autopsy findings. An autopsy revealed a lack of myocardial elasticity, thickening and afferent hypertrophy of the left ventricular wall, dilation, and thinning of the right ventricle (420 g; left ventricular wall, 9 mm; right ventricular wall, 3 mm), and partial rupture of the chorda tendinae. (*A*) Extensive cardiomyocyte nuclear desquamation and necrosis were observed in the left ventricular anterior portion and right ventricular portion. Inflammatory cell infiltration was minimal, and the myocardial tissue was rough, especially on the right ventricular side in the ventricular septum. Oedema of the interstitium was observed with some fibrosis. (Haematoxylin and eosin staining, × 20 magnification, b: left ventricle, c: right ventricle) (*B*, *C*) A small amount of inflammatory cell infiltration was observed in the myocardial tissue. The eosinophil count was also markedly decreased. Lipofuscin granules were observed. (Haematoxylin and eosin staining, × 40 magnification) (*D*).

An autopsy revealed a lack of myocardial elasticity, thickening and afferent hypertrophy of the LV wall, and dilation and thinning of the RV (420 g; LV wall, 9 mm; RV wall, 3 mm). Extensive cardiomyocyte nuclear desquamation and necrosis were observed in the anterior portions of the LV and RV. Inflammatory cell infiltration was minimal, and the myocardial tissue was rough, particularly on the RV side of the ventricular septum. Oedema of the interstitium was observed with some fibrosis (*Figure 3*). With no evidence of malignancy or eosinophilic infiltration in organs other than the heart, idiopathic EM was diagnosed.

## Discussion

EM is a rare inflammatory heart disorder characterized by eosinophilic myocardial infiltration. Although often idiopathic, EM is associated with various conditions, none of which were present in this patient. Interestingly, 14.1% of histologically confirmed cases lacked peripheral blood eosinophilia.^2^ EM diagnosis can be challenging, with 19% of cases diagnosed after autopsy. Biomarkers of inflammation can aid in diagnosis but are absent in over 20% of cases. Endomyocardial biopsy allows definitive EM diagnosis and is considered beneficial.^5^ In this case, the diagnosis of EM was solely based on myocardial biopsy results, which subsequently guided the appropriate treatment.

Fulminant myocarditis, a rare but severe condition, often requires inotropes or mechanical support. An early diagnosis is the key because steroid treatment has shown to enhance cardiac function in some cases.^6^ In our region study, the cumulative risk of death or heart transplantation after 90 days was 29%. Cox regression analysis showed that older age, nonsinus rhythm, low LV wall motion (<40%) on admission, and VT or fibrillation were associated with a worse 90-day survival.^7^ Our patient had all poor prognostic factors, highlighting the difficulty of treatment.

The patient died despite receiving intensive care. Steroid therapy under the management of ECPELLA led to an improvement in LV function and cardiac enzyme levels, but RV dysfunction persisted. RV dysfunction, although relatively uncommon (occurring in 17.8% of cases), is associated with a higher frequency of cardiac events than LV dysfunction alone.^8^ The use of Impella RP may have had the potential to improve outcomes as it assists RV function, but it has not been approved for commercial use in our region.^9^ At autopsy, the myocardial tissue showed only a slight inflammatory cell infiltrate compared to pre-treatment, suggesting that the inflammation had largely been controlled by steroid therapy. However, the myocardial damage was severe and irreversible, suggesting that the initiation of treatment might have been delayed. This case underscores the complexity and severity of eosinophilic fulminant myocarditis and highlights the importance of early diagnosis and treatment.

This case involved a patient with fulminant idiopathic EM who died despite treatment, including mechanical circulatory support. In fulminant myocarditis, early therapeutic interventions, including steroids, should be considered even without pathological findings of EM.

We report an autopsy case of idiopathic fulminant EM with biventricular dysfunction. In fulminant myocarditis, early therapeutic interventions, including steroids, should be considered even without pathologies or findings suggestive of EM, as early pathological diagnosis is vital.

## Lead author biography


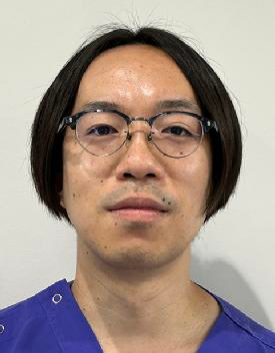


Eiji Koyama is a cardiac intervention specialist who works for Shonan Kamakura General Hospital in Japan. He received his medical degree at the Nihon University in 2017.

## Supplementary Material

ytaf262_Supplementary_Data

## Data Availability

The datasets used and/or analysed during the current study are available from the corresponding author on reasonable request.

## Supplementary material

**Statement of consent:** Informed consent was obtained from the patient in accordance with the Committee on Publication Ethics (COPE) guidelines.

**Funding:** None declared.
